# Analysis of anther transcriptomes to identify genes contributing to meiosis and male gametophyte development in rice

**DOI:** 10.1186/1471-2229-11-78

**Published:** 2011-05-09

**Authors:** Priyanka Deveshwar, William D Bovill, Rita Sharma, Jason A Able, Sanjay Kapoor

**Affiliations:** 1 Interdisciplinary Centre for Plant Genomics and Department of Plant Molecular Biology, University of Delhi, South Campus, New Delhi - 110021, India; 2 Waite Research Institute and School of Agriculture, Food & Wine, The University of Adelaide, Waite Campus, PMB1, Glen Osmond, South Australia 5064, Australia; 3 Current address: Department of Plant Pathology, University of California, Davis, CA, USA

## Abstract

**Background:**

In flowering plants, the anther is the site of male gametophyte development. Two major events in the development of the male germline are meiosis and the asymmetric division in the male gametophyte that gives rise to the vegetative and generative cells, and the following mitotic division in the generative cell that produces two sperm cells. Anther transcriptomes have been analyzed in many plant species at progressive stages of development by using microarray and sequence-by synthesis-technologies to identify genes that regulate anther development. Here we report a comprehensive analysis of rice anther transcriptomes at four distinct stages, focusing on identifying regulatory components that contribute to male meiosis and germline development. Further, these transcriptomes have been compared with the transcriptomes of 10 stages of rice vegetative and seed development to identify genes that express specifically during anther development.

**Results:**

Transcriptome profiling of four stages of anther development in rice including pre-meiotic (PMA), meiotic (MA), anthers at single-celled (SCP) and tri-nucleate pollen (TPA) revealed about 22,000 genes expressing in at least one of the anther developmental stages, with the highest number in MA (18,090) and the lowest (15,465) in TPA. Comparison of these transcriptome profiles to an in-house generated microarray-based transcriptomics database comprising of 10 stages/tissues of vegetative as well as reproductive development in rice resulted in the identification of 1,000 genes specifically expressed in anther stages. From this sub-set, 453 genes were specific to TPA, while 78 and 184 genes were expressed specifically in MA and SCP, respectively. The expression pattern of selected genes has been validated using real time PCR and *in situ *hybridizations. Gene ontology and pathway analysis of stage-specific genes revealed that those encoding transcription factors and components of protein folding, sorting and degradation pathway genes dominated in MA, whereas in TPA, those coding for cell structure and signal transduction components were in abundance. Interestingly, about 50% of the genes with anther-specific expression have not been annotated so far.

**Conclusions:**

Not only have we provided the transcriptome constituents of four landmark stages of anther development in rice but we have also identified genes that express exclusively in these stages. It is likely that many of these candidates may therefore contribute to specific aspects of anther and/or male gametophyte development in rice. In addition, the gene sets that have been produced will assist the plant reproductive community in building a deeper understanding of underlying regulatory networks and in selecting gene candidates for functional validation.

## Background

The anther is the male reproductive organ in flowering plants and is composed of both reproductive and non-reproductive tissues. The reproductive tissue originates as a mass of primary sporogenous cells which are produced from the division of archesporial cells in the L2 layer of anther primordia. These cells divide mitotically to give rise to the microspore mother cells (or meiocytes), that undergo meiosis to produce haploid tetrads of microspores [1]. This reductional division assures genetic diversity in sexual reproduction *via *pairing and recombination between homologous chromosomes. Cytologically, there are more commonalities than differences between the processes of mitosis and meiosis, e.g., condensation of chromosomes, their distinctive alignment at metaphase, followed by separation of sister chromatids/homologous chromosomes at anaphase, grouping of two nucleoids at telophase, and finally cytokinesis that physically partitions the nucleo-cytoplasmic compartments. Besides these similarities, there are a few vital dissimilarities that distinguish these two processes, including pairing and recombination of homologous chromosomes during meiosis (which underlines the basis of genetic diversity). This is followed by segregation of homologues and non-sister chromatids by unipolar attachment of sister kinetochores to spindles, during the first meiotic division. In the last decade, a number of cell division components involved in chromosome condensation, sister chromatid/homologous chromosome cohesion, kinetochore-spindle attachment/alignment, and cytokinesis have been identified. However, we still know very little about the regulatory networks that control the functioning of such components in a mitosis- or meiosis-specific manner.

Unlike in animals, haploid sperm are not produced directly after meiosis in plants. Instead, the haploid microspores are freed from the tetrad by the action of callase, and then divide mitotically twice to produce a three-celled functional male gametophyte known as pollen. The first mitosis is asymmetric which results in two cells of different sizes and with dissimilar fates. The larger vegetative cell occupies most of the pollen space and does not divide further but later, at the time of germination, forms the pollen tube. The smaller generative cell undergoes one more round of mitotic division (symmetrical this time) to produce two sperm cells. One of the sperm cells fertilizes the egg cell in the female gametophyte to form the zygote and the other fuses with the two polar nuclei to form the triploid endosperm. Development and release of mature pollen is dependent on the elaborate coordination of many genes expressed in both non-reproductive as well as reproductive cell layers of the anther. Thus, the anther is a multicellular organ that undergoes complex processes such as cell fate determination [2], cell differentiation, reductional division [3] and cell-cell communication [4].

Our understanding of the genes that regulate developmental aspects of the anther is largely based on information gathered by gene function knockdown approaches, either by mutagenesis or RNA interference (RNAi). Most of the pioneering research has been done in *Arabidopsis *but at the same time many genes have also been identified and characterized in rice revealing gene function deviations or novel gene functions (for reviews, *see *[5,6]). For example, the characterization of an *Arabidopsis EXCESS MICROSPOROCYTES 1 (EXS/EMS1) *orthologue in rice (*MULTIPLE SPROPOROCYTES I *- *MSPI) *and subsequent delineation of its interaction with the *TAPETUM DETERMINANT 1 (TPD1) *rice orthologue (*OsTDL1A*), revealed its novel function in restricting the number of sporogenous cells in the ovule as well as in the anthers [2,7-10].

Although the gene knockout/knockdown approach (in combination with the over/ectopic-expression approach) can enable classification of a particular gene in context of a biological phenomenon, these methods do not provide detailed information about the other components of the regulatory circuitry that are positioned either upstream or downstream in the hierarchy. Building a regulatory network around this nucleation point is often a difficult task that involves a combination of protein-protein, DNA-protein and mutant analysis strategies. However, analysis of transcriptome level perturbations in developmentally or physiologically distinct states may help in the segregation of various molecular contributors into co-expression groups, which could be further analyzed for specific interactions [11,12]. Microarray-based studies carried out in *Arabidopsis *[13], wheat [14] and rice [15] have revealed the complexity of gene expression during stages of anther development by use of high density microarrays. Honys and Twell [13] carried out transcriptome analysis of male gametophyte development in *Arabidopsis *where they identified and categorized microspore-expressed genes on the basis of co-expression profiles. Of particular note is the study conducted by Crismani and co-workers [14], where these authors used wheat Affymetrix GeneChip to monitor the expression dynamics across seven stages of anther development in the complex polyploid, bread wheat. More recently, in rice, distinguishable differences between the tapetum and male gametophyte transcriptomes have been ascertained by using laser micro-dissected cells of specific tissue types [16,17]. Collectively, all these studies highlight the contrast of expression between gametophytic and sporophytic tissues. However, because of the lack of comparison with other tissue/cell-types most of these studies fall short of identifying genes that express specifically in these cell types and, therefore, would almost certainly be playing significant regulatory roles in controlling various aspects that are unique to male gametophyte development.

An objective of the current study was to identify genes that exhibit anther stage-specific expression patterns. To achieve this we performed whole genome microarray analysis on rice anthers isolated at pre-meiotic (PMA), meiotic (MA), single-celled microspore (SCP), and tri-nucleate pollen (TPA) stages of development. Since whole anthers were used in this study, we expected the data to include contributions from all cell types. We performed differential expression analysis to identify genes regulating precise developmental events during anther development. By including transcriptomic data of four vegetative and seed developmental stages/tissue types in the differential expression analysis, we have attempted to identify and segregate expression profiles specifically (preferentially) relevant to the events related to male gametophyte development. These analyses have identified genes that express specifically in PMA, MA, SCP and TPA. Furthermore, the data have also been analyzed for the expression specificities of known meiosis-related genes and those contributing to sperm cell transcriptomes in other systems. Our data therefore provides a firm foundation for future investigations centered on delineating the molecular networks of male meiosis, early gametophyte development and sperm cell differentiation in rice.

## Methods

### Tissue collection and RNA extraction

Wild type rice (*Oryza sativa *subsp. *indica *cv. IR64) was transplanted in fields in mid-June, 2007. Temperature ranged from 35-40°C^max ^and 25-29°C^min^. A constant water supply was available throughout the growing period. Tissue was harvested at different stages of anther development from about 30 to 60 days after transplant. Florets at various stages of development were dissected using a Leica MZ 12.5 (Leica Gmbh, Wetzlar, Germany) dissecting microscope to collect anthers. Anther squashes were prepared from one representative anther in each floret, stained with DAPI, and observed under a fluorescence microscope (DM 5000B, Leica Gmbh, Wetzlar, Germany) to confirm the developmental stage according to Raghvan [18]. Anthers isolated from 8-10 plants were bulked into three biological replicates.

After collection and staging into separate groups containing four developmentally distinct stages [pre-meiotic anther (PMA; from the first identifiable anther like structure to the end of interface), meiotic anther (MA; leptotene to tetrad), anthers with single celled pollen (SCP) and anthers with tri-nucleate pollen (TPA); Table 1], anthers were placed in Trizol reagent (Invitrogen, CA, USA) and kept at -70°C until RNA isolation. High quality RNA, assessed by a bio-analyzer (Agilent, CA, USA), was used for hybridization experiments with the 57K Rice Genome Array (Affymetrix, CA, USA).

**Table 1:** Classification of rice panicles and florets for categorization of anther development stages

	Anther Development			
	
	(PMA) Pre-meiotic anther	(MA) Meiotic anther	(SCP) Anther with single celled pollen	(TPA) Anther with tri-nucleate pollen
				
Anther development stage [47]	Stage 3-5	Stage 6-8	Stage 9-10	Stage 12-14
Anther length (mm)	0.35-0.45	0.50-0.85	0.90-0.95	2.0-2.5
Floret length (mm)	1.5-2.5	3.5-6.0	7.0-7.5	>8.0
Panicle length (cm)	1.0-5.0	6.0-11.0	8.0-15.0	25.0-30.0

*Note*: Panicle, floret and anther length indexing is standardized only for IR64 cultivar of *Oryza sativa *subsp. *indica*, and may vary in different cultivars of rice

### Microarray experiments

A total of 3 μg of total RNA isolated from anthers was amplified and labeled using a one-cycle target labeling kit (Affymetrix, CA, USA). Target preparation, hybridization, washing, staining and scanning of the chips were done according to the manufacturer's protocol. GeneChip^® ^Operating Software 1.2.1 (GCOS) was used for washing and staining of the chips in a Fluidics Station 450 (Affymetrix, CA, USA) and scanned with a Scanner 3300 (Affymetrix, CA, USA). Three biological replicates processed for each stage with overall correlation co-efficient values of more than 0.97 were further used for the final data analysis, which underlines the high reproducibility and reliability of the microarray data.

### Microarray data analysis

CEL files for four anther development stages generated by GCOS were transferred to ArrayAssist ver. 5.5 (Stratagene, CA, USA) microarray data analysis software for analyses. A combined project was made where CEL files of the four anther stages, as well as those for mature leaf, Y-leaf, root, 7-day-old seedling, shoot apical meristem (SAM; meristematic tissue isolated from the apex of the shoot from plants in which more than half of the tillers already had panicles) and five stages of seed development (S1, S2, S3, S4 and S5), have been deposited to the Gene Expression Omnibus (GEO; http://www.ncbi.nlm.nih.gov/geo/; accession numbers GSE6893 and GSE6901).

The rice Affymetrix GeneChip^® ^contains 57,381 probe-sets, however, not all of the probe-sets correspond to annotated genes, and in some instances more than one probe-set corresponds to annotated genes. Therefore, in order to identify the unique probe-sets that correspond to annotated genes, the MSU Rice Pseudomolecule (ftp://ftp.plantbiology.msu.edu/pub/data/Eukaryotic_Projects/o_sativa/annotation_dbs/) version 5, KOME (http://cdna01.dna.affrc.go.jp/cDNA/) and NCBI (http://www.ncbi.nlm.nih.gov/) databases were used, with the probe-set list manually curated. Consequently, a total of 37,927 probe-sets were identified as unique non-redundant probe-set IDs (after removing hybridization controls, transposable element (TE) related genes, redundant probe-sets and probe-sets without a corresponding locus in the databases mentioned above). All subsequent expression analysis was carried out on this reduced dataset. The MAS5 algorithm was applied (with default parameters) to identify genes that could be classified as expressed or non-expressed. 66% present calls in a triplicate (as PPP, PPA or PMM) dataset were kept as minimum criteria for a gene being 'expressed' or otherwise 'non-expressed'. The microarray data was normalized using the GC-RMA algorithm followed by log_2 _transformation. To identify differentially expressed genes, one-way Analysis of Variance (ANOVA) was performed on the four anther development stages with the Benjamini Hochberg correction [19]. Further, a stringent statistical criterion of at least a 2-fold change at a p-value ≤0.005 was used for gene selection. Cluster analysis was performed using the *K-means *clustering algorithm of ArrayAssist (Stratagene, La Jolla, CA, USA). All the heat-maps were made using GC-RMA log transformed sample averages.

Expression values of probe-sets of *Magnoporthe *genes present on the chip were used as a criterion to define "absent" genes (Additional File 1) since their signal value should represent the background signal. Average of the median for those genes plus 5 i.e., 10 GC-RMA value was put as the upper limit for a gene to be called 'absent'. Annotations for functional alignment of genes were retrieved from Osa1 Rice Genome Annotation Project release 6 (RGAP: http://rice.plantbiology.msu.edu/).

### Identification of putative orthologues in rice

We have previously described the identification of putative rice orthologues of meiotic genes [20]. Briefly, the sequences of *Saccharomyces cerevisiae *and *Arabidopsis thaliana *genes involved in double strand break (DSB) formation, recombination, synaptonemal complex assembly, chromosome pairing and DNA mismatch repair were used as queries for TBLASTX analysis against all green plants at The Institute for Genomic Research's (TIGR) Plant Transcript Assembly (TA) database. A significance value of >E^-20 ^from the TBLASTX analysis was used to identify putative orthologues in wheat, rice and barley. The rice TA IDs for meiotic gene orthologues [20] were used to identify the corresponding rice Osa1 loci (MSU Rice Genome Annotation (Osa1) Release 6.1; http://rice.plantbiology.msu.edu) and their respective Affymetrix probe-sets, which were used for expression analysis. For the identification of sperm-expressed genes, cDNA and EST sequences of *Arabidopsis*, maize and lily were downloaded from TAIR (http://www.arabidopsis.org/) and NCBI (http://www.ncbi.nlm.nih.gov/). These sequences were used as queries for BLASTx against a local database made with the Osa1 Release 6.1 Rice proteins using BIOEDIT software (http://www.mbio.ncsu.edu/BioEdit/bioedit.html), with a significance value of > E^-20 ^used for identifying rice orthologues (Additional File 2).

### Real-time quantitative PCR (Q-PCR)

cDNA for the real-time reactions were synthesized using the same RNA samples that were used for microarrays. Real-time PCR primer designing, reactions and calculations were carried out as described previously [21]. Primers used in the experiment are listed in Additional File 3.

### In situ hybridizations

Florets were fixed in FAA (10% formaldehyde, 5% acetic acid and 50% ethanol) for 24 hours at 4°C and then dehydrated in a graded ethanol series followed by a tertiary butanol series, before placing in paraplast plus (Sigma Aldrich). Paraplast embedded florets were sectioned by using a Leica RM2245 rotary microtome producing 8 μm thick sections that were placed on Poly-L-Lysine coated slides (Polysciences Inc.). Approximately 200 bp sequences from the genes LOC_Os04g52550 and LOC_Os01g70440, were amplified using primers (forward 5'-CATGTTCTTCCTCTGACGACA-3' and reverse 5'-GACACGGACAAAAATTTACTATGG-3') and (forward 5'-CTCCACCTCGCTCTGATTAA-3' and reverse 5'-TCATTTCAATGCAGTACAGGC-3'), respectively. These cloned products were then ligated into the pGEMT-Easy vector (Promega). The clones were linearized with *Sal *I and *Nco *I enzymes for *in vitro *transcription of digoxinin labeled RNA probes with T7 and SP6 RNA polymerase, respectively, according to the manufacturer's instructions (Roche). The *in situ *pre-treatment and hybridization steps were essentially carried out as described [22]. Immunological detection was carried out using the Roche DIG detection kit, following the manufacturer's protocol. Sections were mounted in DPX mounting medium and observed under the microscope (DM 5000B, Leica Gmbh, Wetzlar, Germany).

## Results

### Development-dependent changes in the anther transcriptome

Transcriptome profiling of anther development required isolation of anthers at landmark stages of development, i.e., pre-meiosis (PMA), meiosis (MA), immediately after meiosis where single-celled microspores are released from tetrads (SCP) and mature anthers with tri-nucleate pollen (TPA) just prior to dehiscence. For this, the rice florets were initially broadly classified on the basis of their size and then one anther from each floret was microscopically examined to confirm the stage of male gametophyte development by staining with DAPI before staging the rest into one of the four classes specified above (Table 1). Microarray data from the three replicates of each stage exhibited correlation co-efficients of 0.99 (PMA), 0.99 (MA), 0.99 (SCP) and 0.97 (TPA). Scatter plot analysis was performed to analyze the extent of transcriptome level variations between the four anther stages (Additional File 4). Interestingly, PMA, MA and SCP showed high correlation values between 0.92-0.96, however, TPA was found to be markedly different in its transcript constitution from the other stages of anther development, with correlation co-efficients ranging between 0.77 and 0.79. This difference was also reflected in the number of differentially (2-fold at p-value ≤ 0.005) regulated genes (7219-8318 between TPA and other anther stages). To determine the extent of transcriptome level changes that are required for anthers to differentiate from the undifferentiated meristematic cells, the PMA transcriptome was compared with that of the shoot apical meristem (SAM). The SAM and PMA showed significant correlation (0.94), which gradually declined with the progression of anther development to 0.90 (SAM:MA), 0.87 (SAM:SCP) and 0.73 (SAM:TPA).

**Figure 1:**
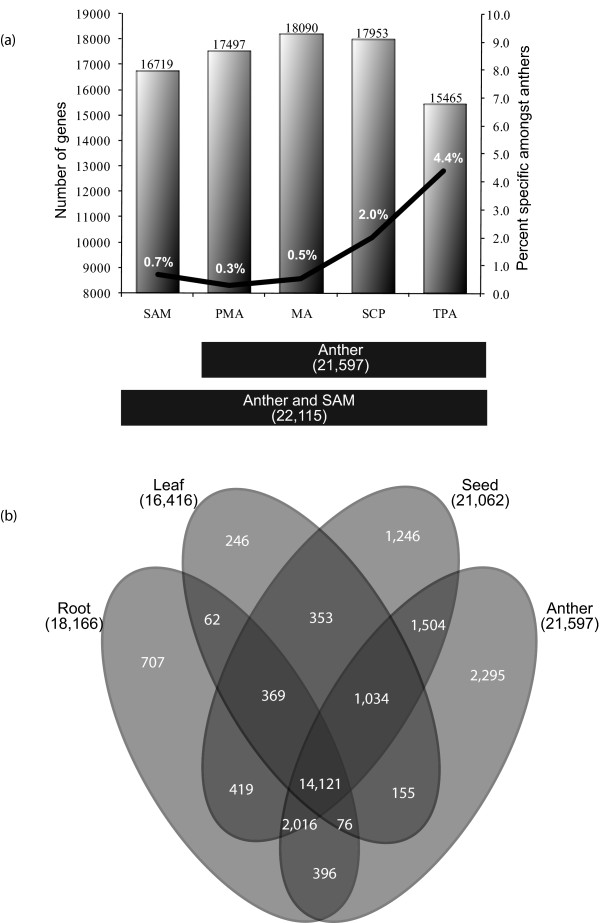
**Transcriptome profile of anther development**. (a) Anther development transcript sizes overlaid with a line graph depicting the percentage of specifically expressed genes in individual stages. The figure highlights that the meiotic anthers have the largest transcriptome, whereas, anthers at the tri-nucleate stage of pollen development show a comparatively smaller transcriptome, but with the largest proportion of specific genes. (b) Venn diagrams showing the constitution of vegetative tissues (leaf and root), seed and anther transcriptomes with component overlaps amongst them.

The oligonucleotide probes on the rice Affymetrix Genome Array represent 37,927 unique genes including 33,813 gene loci mapped in MSU Rice Genome Annotation Release 6 and 4,114 unique, but unmapped, cDNA/ESTs (KOME and NCBI). This represents 93.5% of the latest estimates of 40,577 non-TE-related protein-coding genes on the rice pseudomolecules. To define the extent of the anther transcriptome, the expressed genes were differentiated from the non-expressed genes (*see *Materials and Methods). Consequently, 21,597 genes were identified as expressed in at least one stage of anther development (Figure 1a). MAS5 detection calls and their p-values are given in Additional File 5. The MA and SCP stages were found to express the maximum number of genes, i.e., 18,090 and 17,953, respectively. Number of genes specifically present amongst anthers was identified as those where expression in all the other anther stages except one had GC-RMA expression value less than 10 (*see *Materials and Methods). The TPA transcriptome was the smallest with 15,465 expressed genes but it represented the most diverse transcriptome with the largest proportion (4.4%) of genes expressed specifically at this developmental stage amongst anthers. The proportion of specifically expressed genes was found to be 2.0%, 0.5% and 0.3% in SCP, MA and PMA, respectively.

The cumulative anther transcriptome was compared with the previously generated transcriptomes of root, leaf and five stages of seed development of the same rice cultivar [21,23] to identify the extent of overlap between various transcriptomes (Figure 1b). In total, 14,121 genes express in all the stages analyzed, suggesting their involvement in housekeeping functions or general metabolism. This analysis also highlighted that anthers have the largest (21,597 genes) and the most diverse transcriptome of all the stages analyzed, as expression of 2,295 (10.6%) genes was unique to anthers. In comparison, the numbers of uniquely expressed genes in roots, leaves and seeds were 707, 246 and 1,246, respectively. Besides identifying 14,121 commonly expressed genes between all four developmental stages, the anther transcriptome shared maximum similarity to that of the seed transcriptome with 4,554 commonly expressed genes in anther and seed stages. However, a much lower level of similarity between the anther and root (2,488), and anther and leaf (1,265) transcriptomes was observed.

### Co-regulated clusters of differentially expressed genes

**Figure 2:**
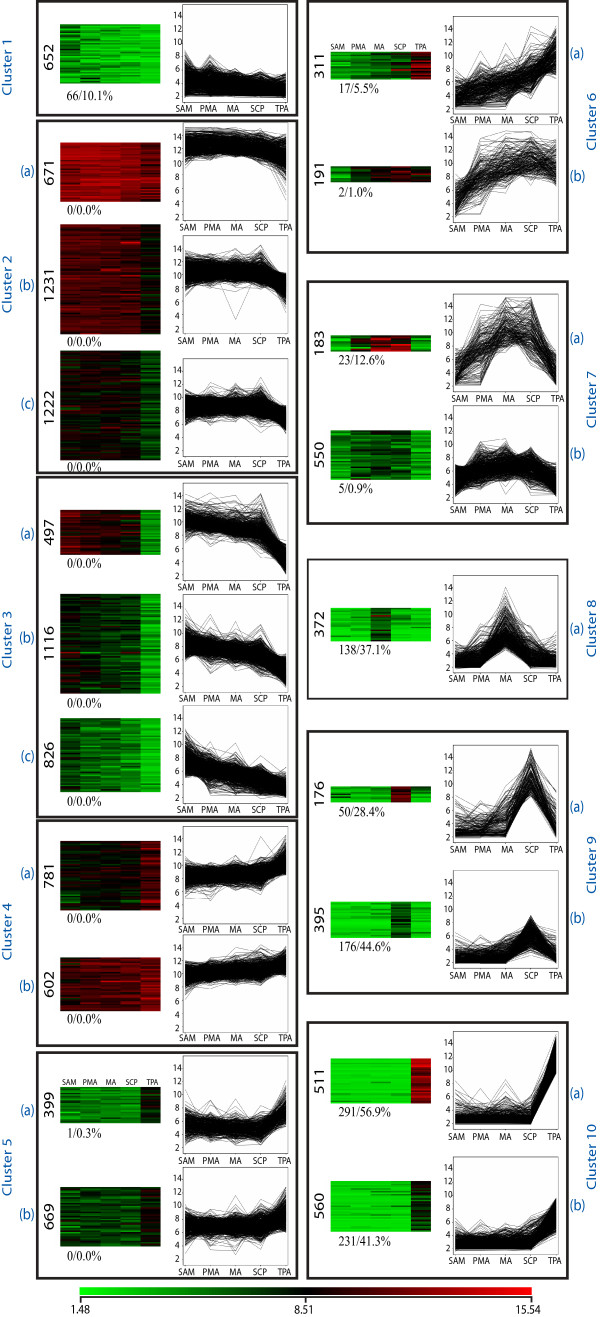
**Gene expression patterns of differentially expressed genes in SAM and the four stages of anther development (PMA, MA, SCP, TPA) categorized into 20 groups using the *K-means *clustering tool**. Groups with similar expression patterns but different expression amplitudes have been grouped together to make 10 clusters. The normalized log transformed signal values were plotted for each of the five stages. The number of genes in the clusters is indicated along the side of the heatmap. The percentage of anther-specific genes in each cluster is specified at the lower left side of the heatmap.

To identify genes with similar expression profiles during anther development, the normalized expression data was subjected to one-way ANOVA that resulted in the selection of 14,672 differentially expressed genes at a p-value ≤0.005. Using a cut-off of 2-fold change in expression in any stage of anther development further filtered these genes to 11,915 (Additional File 6). Using *K-means *clustering, these genes could be clustered into 10 major groups, which were further categorized into sub-groups depending on the amplitude of expression (Figure 2). Clusters 2 to 5 consisted of 8,014 (67.3%) differentially expressed genes expressing in all stages of anther development. Of these, only one gene was found to be specific to anther stages. Genes in these clusters either showed up (cluster 4 and 5) or down regulation (clusters 2 and 3) in TPA, while in other stages the difference in expression of these genes is not as significant. In contrast, the 733 (6.2%) genes in cluster 7 showed high expression in PMA, MA and SCP; 571 (4.8%) genes in cluster 9 were activated specifically in SCP, while clusters 8 (372 genes; 3.1%) and 10 (1,071 genes; 9.0%) exhibited MA- and TPA-preferential expression profiles, respectively.

**Figure 3:**
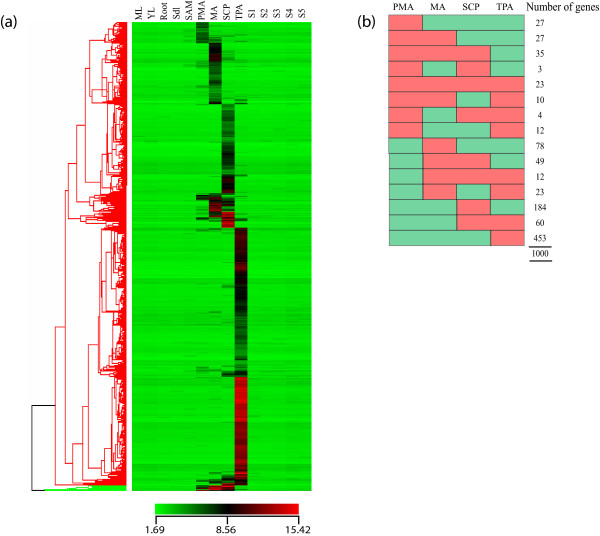
**Expression profiles of specifically expressed genes in anthers**. (a) Hierarchical cluster diagram representing expression patterns of 1000 genes that show transcript accumulation in at least one of the four stages of anther development and undetectable expression in any of the vegetative (ML, mature leaf; YL, Y-leaf; Root; SDL, 7-day-old seedling) or seed development stages (S1-S5; encompassing 0-30 days of seed development after pollination). (b) A diagrammatic representation of the anther-specific expression profiles with the number of genes under each expression profile.

For the identification of specifically expressed genes during anther development, five vegetative stages (mature leaf, Y leaf, root, 7 day old seedling and SAM) and five stages of seed development (S1, S2, S3, S4, S5) were compared with anther stages. From the 11,915 differentially expressed genes (from Figure 2), those with GC-RMA normalized signal values less than or equal to 10 in vegetative and seed stages were filtered out (*see *Materials and Methods for criteria on 'absent' genes). Genes obtained were further filtered by identifying those with at least a 2-fold higher signal value in any of the anther stages than the highest value in the vegetative or seed stages (i.e. these candidates would have at least a 20 GC-RMA signal value). After such stringent filtering 1,000 anther-specific genes were identified (Figure 3). Forty-five percent (45.3%) of them were only specifically expressed in TPA, further emphasizing the distinctness of this stage. SCP and MA have only 18.4% and 7.8% of the specifically expressing genes respectively, while PMA has a low share of stage specificity with 2.7% representation. Notably, those specifically expressed in PMA have lower expression compared to other anther stages.

Percentages of anther specific genes were calculated for each of the k-means clusters (Figure 2). Interestingly, expression of 33.3% (914 genes) of the 2,747 genes in clusters 7 to 10 was found to be specific to anthers. Of these 914 genes, 138 (15.1%) were specific to meiotic anthers, 226 (24.7%) to anthers at the SCP stage, while the largest group was expressed specifically at the TPA stage (522 genes; 57.1%) (*see *Additional File 6).

The differentially expressed genes in each of the 10 clusters were assigned to 19 functional categories and those that could not be affiliated to any of these categories or that have not been annotated as yet were categorized as 'Others' (approximately 34%; Table 2). Cluster-wise over representation of the number of genes by 20% (taken arbitrarily as a measure of predominance) of their overall percentage in individual functional categories has been highlighted to facilitate better visual interpretation of the data (Table 2). Genes involved in protein metabolism, involving folding, sorting and degradation (6.9%), signal transduction (8.3%) and transcription factors (7.1%) constitute three major functional categories of differentially expressed genes during anther development. Clusters 1, 2 and 3, which exhibited down-regulatory trends from SAM to TPA (*see *Figure 2), were dominated generally by transcription factor, chromatin remodeling, RNA metabolism, translation- and cell cycle-related genes. Expression profiles in clusters 6b and 7, showing up-regulation in MA and SCP followed by down-regulation in TPA, coincide with the pattern of tapetum development. Coincidently, the genes exhibiting these profiles were found to have over-representation of those involved in carbohydrate, energy and lipid metabolism, along with those involved in transporter activities and vesicular trafficking. Cluster 10, which represents TPA specific expression profiles, had an over-representation of genes involved in cell structure, secondary metabolism, transporter activity and signal transduction.

**Table 2:** Association of differentially expressed genes in co-expression clusters (see Figure 2) with GO functional categories

	**Percentage of transcripts classified in co-expression profiles in Figure 2**.										
	
Functional Categories	High to low			Low to high		PMA, MA, SCP, TPA	PMA, MA, SCP	MA	SCP	TPA	
	
	1	2	3	4	5	6	7	8	9	10	Total
Amino acid metabolism	0.6	***1.6***	1.1	1.4	**1.8**	0.8	1.1	0.3	0.2	0.6	1.2
Carbohydrate metabolism	0.8	2.0	1.2	***4.4***	1.4	***3.4***	***2.5***	1.1	1.4	1.6	2.0
Catalytic activity	1.7	2.7	2.4	***4.8***	3.8	***6.8***	***5.7***	4.0	3.0	3.9	3.5
Cell cycle	2.3	3.3	***6.1***	2.1	2.3	1.2	1.2	1.6	0.7	1.3	3.0
Cell structure	1.7	2.0	***3.0***	1.7	1.4	2.4	2.6	2.2	2.5	***5.4***	2.5
Chromatin remodeling	1.7	***2.7***	***2.5***	1.3	1.0	1.0	0.7	0.5	0.4	0.6	1.7
Energy metabolism	1.5	***3.6***	1.6	***4.7***	1.8	***3.4***	3.0	2.7	2.5	1.3	2.7
Lipid metabolism	1.1	2.2	2.5	***3.6***	3.0	***6.6***	***4.4***	3.0	***4.6***	2.6	2.9
Nucleotide metabolism	0.6	1.3	1.6	***1.5***	1.1	0.0	0.7	0.0	0.7	0.4	1.1
Protein-protein interaction	2.3	3.3	2.7	***4.4***	2.3	2.8	2.6	2.7	***4.0***	2.1	3.0
Protein metabolism	5.7	7.9	6.3	6.8	7.3	4.4	6.3	***8.9***	***11.6***	4.0	**6.9**
RNA metabolism	***7.7***	***7.5***	***7.4***	2.2	3.8	1.2	1.9	1.1	1.4	1.0	4.8
Secondary metabolism	1.8	0.6	0.9	0.9	1.2	***3.8***	***4.0***	***3.2***	***5.3***	***2.7***	1.7
Signal transduction	6.6	7.5	8.8	9.0	8.2	7.8	9.3	7.8	6.8	***10.5***	**8.3**
Stress	4.4	3.1	3.0	3.5	3.7	***6.6***	***6.5***	4.0	***5.1***	4.6	3.8
Transcription factors	***8.9***	8.0	7.7	5.3	5.1	7.0	7.6	***8.9***	6.1	5.7	**7.1**
Translation	1.1	***8.8***	2.8	1.5	1.3	0.2	1.0	0.0	0.7	0.2	3.4
Transporters	2.3	4.1	3.5	***4.8***	***5.5***	***7.8***	4.4	4.0	***5.4***	***6.2***	4.5
Vesicular trafficking	0.3	***2.9***	1.0	***5.0***	2.4	***2.6***	0.7	0.0	0.4	2.2	2.1
	
Others	46.9	25.1	33.9	31.1	41.3	30.5	34.0	44.1	37.5	43.1	33.8

Genes in each cluster	652	3124	2439	1383	1068	502	733	372	571	1071	11915

The total representation of genes (% values) of three major functional categories (besides 'Others') is shown in bold & underlined text. Over-representation of genes in each functional category by more than 20% of their overall representation in individual clusters is highlighted with bold and italicized letters.

### Validation of specific expression profiles by Q-PCR and in situ hybridizations

**Figure 4:**
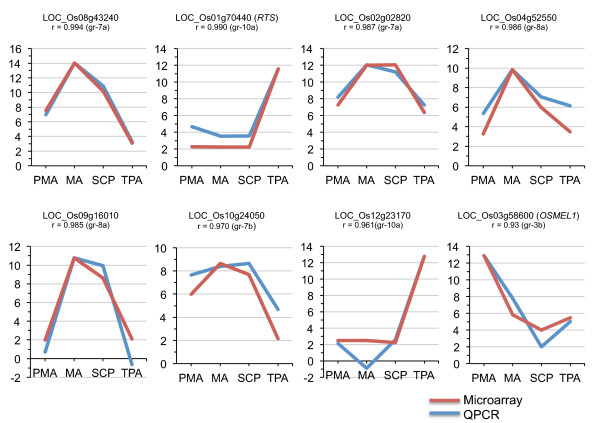
**Q-PCR analysis of eight genes showing anther developmental stage-specific expression and its correlation with microarray data**. Three biological replicates were taken for both Q-PCR and microarray analysis. The Y axis represents normalized log_2 _transformed expression values obtained using microarray analysis and log_2 _transformed relative transcript amount obtained by Q-PCR. The Q-PCR data has been scaled such that the maximum expression value of Q-PCR equals that of the maximum value of the microarray to ease profile matching. Gene locus IDs and their affiliation to the co-expression groups shown in Figure 3 are mentioned. The correlation co-efficient (r) between the two expression profiles is also indicated. Expression of 18S rRNA was used as an internal control to normalize the Q-PCR data. PMA; pre-meiotic anthers, MA; meiotic anthers, SCP; anthers with single-celled pollen, TPA; tri-nucleate pollen containing anthers.

To validate the microarray data, eight genes showing specific expression in one or more stages of anther development were selected for real-time/quantitative PCR analysis (Figure 4). These include: one gene from cluster 3b exhibiting PMA specific expression; two genes from cluster 7a and one gene from cluster 7b with high and low expression, respectively, in MA and SCP; two from cluster 8a with MA preferential expression; and two genes from cluster 10a with expression mainly in the TPA. Two of the selected genes have been previously characterized and their reported expression profiles also matched with our analysis (*OsMEL1 *[24], *RTS *[25]). Overall gene expression as identified by the microarray experiments, exhibited a high degree of similarity with that obtained from the Q-PCR analyses with a correlation co-efficient (r) greater than 0.9, thereby indicating the reliability and robustness of the microarray data.

**Figure 5:**
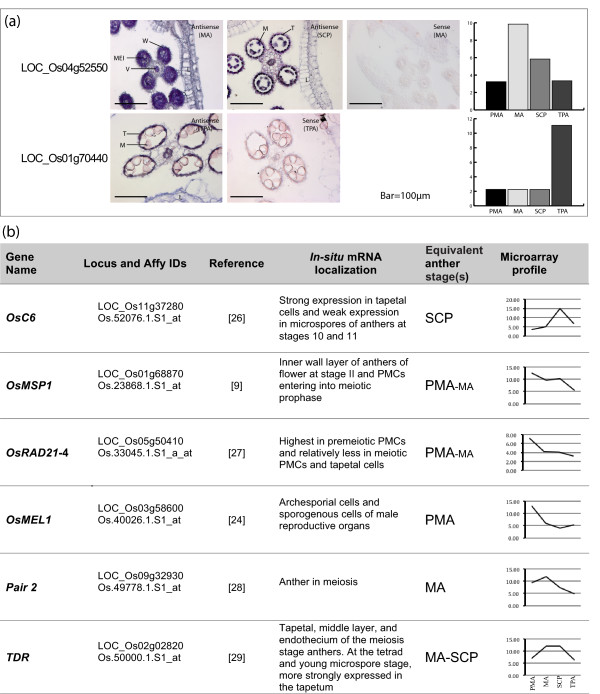
**Validation of microarray data by *in-situ *hybridization**. (a) *In-situ *localization of transcripts corresponding to the genes LOC_Os04g52550 and LOC_Os01g70440 in rice florets (MA, SCP and TPA stages as marked). Corresponding microarray-based expression profiles of these two genes are also shown as bar graphs for comparison. W, wall layers; V, vascular tissue; T, tapetum; M, microspores; MEI, meiocytes; L, lemma. (b) A compilation of *in-situ *localization analyses for six genes using published literature and their correlation with anther preferential expression profiles as revealed by the microarray analysis described in this paper. The log_2 _normalized expression values were used to represent the gene specific microarray profiles.

Further, we validated our microarray expression results by doing *in situ *hybridization of two of the genes already validated by Q-PCR (Figure 5a). The transcripts from LOC_Os04g52550, which codes for an argonaute protein, were found to localize in the meiocytes as well as wall layers of meiotic anthers. Later in development (SCP stage), the expression was found to be restricted to the tapetum, microspores and vascular tissue in the connective. LOC_Os01g70440, coding for a LEM-1 family protein, exhibited expression in the tapetal layer of anthers at tri-nucleate stage with no expression in the pollen grains. The expression of both the genes was restricted to anthers as no expression was seen in lemma and palea (Figure 5a). We also scanned the literature for *in situ *experiments where we could correlate our anther-specific or anther-preferential expression with that reported previously. A summary of expression domains of six such genes coding for OsC6 [26], OsMSP1 [9], OsRAD21-4 [27], OsMEL1 [24], PAIR2 [28] and TDR [29] and their correlation with the microarray expression profiles obtained from our dataset is shown in Figure 5b. The *in situ *expression patterns of two genes analyzed here and the six previously reported, show good correlation with our microarray based profiles and subsequent differential expression analysis.

### Developmental stage-wise activation/up-regulation of genes

**Figure 6:**
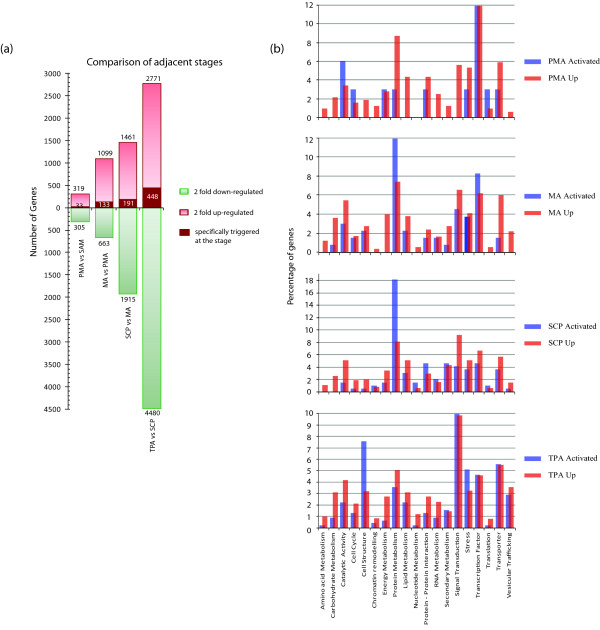
**Analysis of gene activation during anther development**. The transcriptomes of all four anther stages were compared to their preceding stage of development. SAM has been used as the reference for PMA. (a) The number of genes up- or down-regulated ≥ 2-fold at p-value ≤ 0.005 are plotted on the graph. Amongst the up-regulated genes, the numbers that have no detectable expression (GC-RMA value ≤ 10) in any of their previous anther stages as well as non-anther stages have been annotated in maroon boxes in the individual columns as specifically 'triggered'. While such candidates may have expression in later anther stages, expression first appears in that particular anther stage. (b) A bar graph highlighting the distribution of the up-regulated and specifically-triggered genes at individual stages of development into functional categories based on GO annotations.

As anther development progresses from PMA to TPA, a number of processes are accomplished in a sequential manner. By comparing gene expression between two adjacent stages of anther development, we aimed to identify the molecular components involved in switching from one phase of development to the next. The results of this comparative analysis where differences in expression between SAM:PMA, PMA:MA, MA:SCP, and SCP:TPA stages were analyzed by setting the criteria of 2-fold change at a p-value ≤0.005 are shown in Figure 6a. Only a small proportion of genes (624), were found to be differentially activated (319) or down-regulated (305) in PMA when compared to SAM. However the number of differentially expressed genes steadily increased to 1,762 in MA, 3,376 in SCP and 7,251 in TPA in relation to their respective previous stage of development. A greater number of genes were up-regulated in comparison to those down-regulated in PMA and MA, however, this trend reversed in SCP and TPA where a larger proportion of genes showed down-regulation (Figure 6a). This finding might point towards a major post-meiotic switching of gene expression from the sporophytic to the gametophytic mode.

The stage-wise up-regulated genes during progression of anther development were further mined for those that were specifically activated in a particular stage (Figure 6a). For this, specific genes with no detectable expression in any previous anther stage were considered as specifically activated/triggered. Interestingly, only 33 genes (that is, 10.3% of 320 PMA up-regulated genes) were found to be triggered in PMA. The percentage of specifically activated genes ranged between 12 to 16% of the total up-regulated genes in MA, SCP and TPA vis-à-vis their respective previous stage of development, with the number in the respective stages being 133, 191 and 448. Functional association of stage-wise activated and 2 fold up-regulated genes based on Gene Ontology (GO) annotations highlighted the molecular processes/components involved (Figure 6b). Major perturbations in transcript abundance were observed in genes coding for transcription factors, signal transduction and cell structure components, catalytic activity and those involved in the function of protein folding, sorting and degradation. A significant number (45) of genes coding for signal transduction components were specifically activated in TPA, which may contribute to the pollen-specific transcriptome involved in pollen-pistil interactions and pollen tube growth. The largest numbers of genes involved in protein metabolism were triggered in the SCP stage, which coincided with the most active phase of tapetal cells and their degeneration. Out of the 88 cell structure related genes up regulated in TPA, 34 were specifically triggered at this stage that comprises 7.6% of the TPA triggered genes. This suggests most of the up-regulated cytoskeletal genes may have a TPA specific function; most likely in pollen germination.

### Expression dynamics of meiosis-related genes

**Figure 7:**
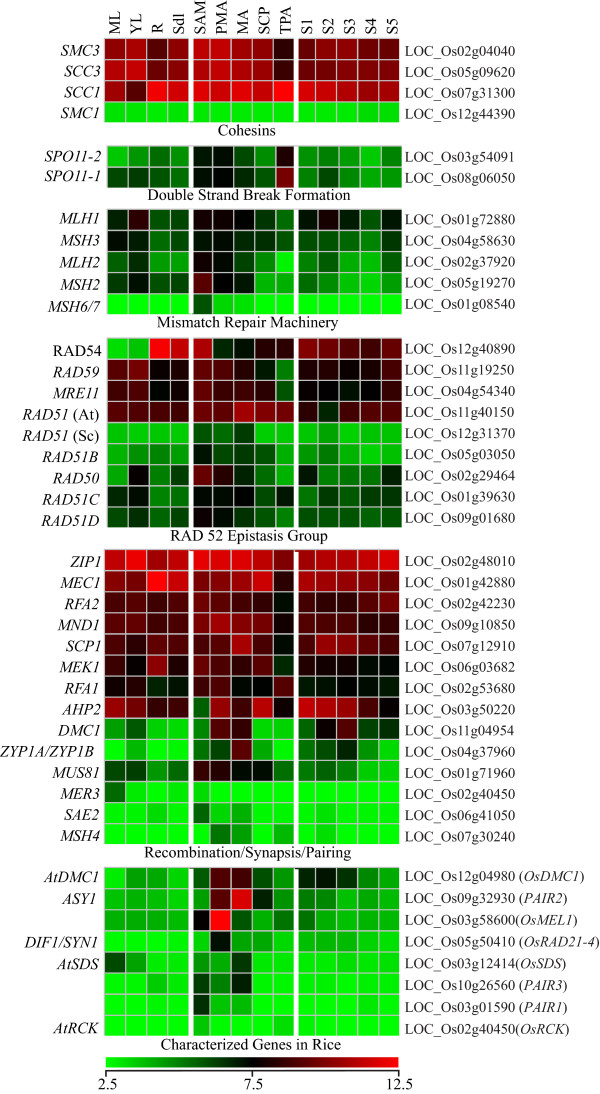
**Expression profiles of putative homologues of known meiosis related genes in yeast and/or *Arabidopsis *that were identified by sequence similarity searches (*see reference ***[20]) **during various developmental stages in rice**. Gene names as taken from the respective sources are shown on the left, while locus IDs of putative homologues in rice are given on the right side of each expression profile. The lowermost panel shows the expression profiles of genes whose functional association in rice has been validated.

The functional conservation of meiosis between eukaryotes can be exploited to identify new candidates for meiotic regulation in rice. We have previously compiled a database of yeast and *Arabidopsis *genes involved in meiosis, and identified putative orthologues in the rice, wheat, and barley genomes [12]. The expression of the rice homologues, identified by tBLASTx, showed that several of these have specific expression/significant up-regulation in anthers, though the majority were also expressed in other tissues/stages of development (Figure 7). Four of the five annotated cohesin genes showed similar expression levels in most tissues, but among these *SMC3 *and *SCC3 *had reduced expression in roots and TPA. The double strand break linked genes *SPO11-1 *and *SPO11-2*, though expressed at relatively lower levels, showed specifically higher transcript accumulation in PMA, MA and TPA. Some components of the mismatch repair machinery (*MSH2, MSH6/7*), RAD52 epistasis group (At*RAD51*, Sc*RAD51, RAD51B, RAD50*) and those involved in recombination/synapsis (*SCP1, MND1, DMC1, ZYP1A/1B, MUS81*) also exhibited higher transcript accumulation in stages of anther development. The yeast meiosis-related genes described above were also analyzed for their expression profiles during vegetative, pre-meiotic and stages after induction of meiosis in yeast (Additional File 7), utilizing the microarray data (GEO Accession no. GSE18256) as described [30]. Of 21 genes analyzed, 11 showed uniform expression in vegetative as well as sporulation stages, while the remaining 10 genes were up regulated by at least two folds during meiosis in yeast (written in red in Additional File 7). Most of the rice orthologues of meiosis-related yeast genes were also found to show enhanced transcript accumulation in anther stages.

Functions for only a few meiosis-related genes, selected on the basis of homology to known genes in other systems or meiosis-affecting mutant phenotypes, have been validated in rice. These genes, with the exception of *AtRCK*, exhibit a characteristic meiotic anther specific expression profile (Figure 7, lowermost panel). Our co-expression analysis revealed two cluster (7 and 8; containing 1105 genes) exhibiting meiotic anther preferential expression profiles (Figure 2). Included in this list are *ZEP1 *[31], *DMC1 *[32]*and *MEL2 [33] which have been implicated in transition from mitotic to meiotic cell division and synapsis of homologous chromosomes. The genes in this cluster, therefore, could be a valuable resource for mining other components of meiotic machinery and meiosis related regulatory networks.

### Proportion of putative sperm cell expressed genes in the TPA transcriptome

**Figure 8:**
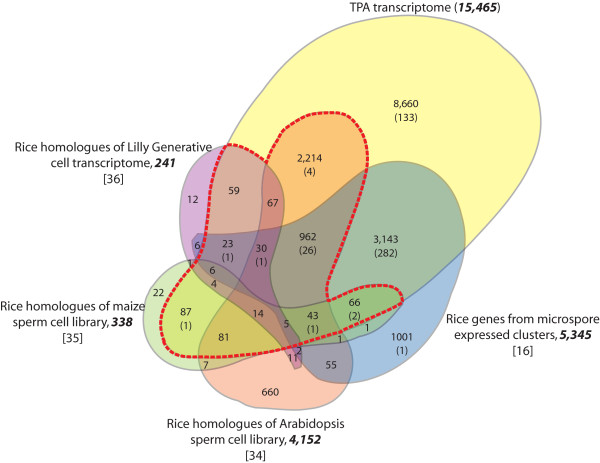
**Identification of putative male gamete transcripts in rice**. The Venn diagram shows overlap between genes that were identified as being present in TPA with microspore preferential genes [16], and homologues found by sequence similarity in the *Arabidopsis *sperm cell transcriptome [34], maize sperm cell ESTs [35] and the lily generative cell transcriptome [36]. The number of genes from the respective transcriptomes that could be mapped on the Rice Genome Array are bold and in italics, while the number of genes that are specifically expressed in the rice TPA transcriptome are indicated in parentheses. The red dashed line constitutes the total number of rice homologues (excluding those in parentheses) that contribute to the putative sperm cell transcriptome in rice which have been identified from the other systems examined.

With the aim of identifying genes contributing to sperm cell transcriptome in rice, we performed a comparative analysis of *Arabidopsis*, maize and lily sperm cell/generative cell expressed genes with the TPA transcriptome [34-36]. We then complemented this analysis by overlaying information on genes with specific expression in rice anthers in order to determine sperm cell-specific genes from the TPA transcriptome. BLASTx analysis identified rice homologues for 338 genes from the maize sperm cell, 3,152 from the *Arabidopsis *sperm cell, and 241 from the lily generative cell transcriptome that were represented on the Rice Genome Array (Additional File 2). Hobo et al., [16] identified 28,141 anther-expressed genes in rice and classified them into 20 clusters based on co-expression profiles; five of which included genes with expression in rice bi-cellular and tri-cellular microspores. The 5,345 genes in these five clusters were also included in this analysis (Additional File 2). Rice homologues of 90.5% maize, 86.7% lily and 82.2% *Arabidopsis *germline-expressed genes were represented in the rice TPA transcriptome. Even the sperm-cell transcriptome datasets obtained from maize and *Arabidopsis *that represent two evolutionary diverse plant groups (monocots and dicots), had proportional representation in the rice TPA transcriptome (Figure 8). However, when the maize and *Arabidopsis *sperm cell transcriptomes were compared with each other, only 151 genes were found to be common, amounting to 44.7% and 3.6% of their respective transcriptomes (Additional File 2). In all, 3,662 rice homologues of sperm cell expressed genes in other systems were identified which would comprise the putative sperm cell transcriptome of rice (Figure 8, indicated by the total number of transcripts delineated within the red dashed line).

## Discussion

The microarray data presented here forms a robust platform for the studies on developmental and molecular aspects of male gametophyte development in rice and in cereals at large. A high degree of correlation obtained between the three biological replicates for all stages investigated underlines the reproducibility and strength of the data, which has also been validated by Q-PCR and *in-situ *hybridization analyses. The MAS 5.0 based present calls representing the size of the transcriptomes (15,465 - 18,090) examined, was found to be significantly higher in comparison to a recent sequencing-by-synthesis based analysis of rice anther transcriptomes in which about 3000 - 12000 distinct transcripts were detected in individual stages of anther development [37]. On the other hand it was much less when compared to another recent study of the rice anther transcriptome using the same platform (Affymetrix) [38] in which 30,186 - 28,280 probe-sets were reported. The difference could have resulted from the fact that we have used a more refined sub-set of genes where the redundant probe-set IDs and genes coding for transposable elements (TEs) were removed. The size of maize anther transcriptomes (based on 44 K maize array), however, was found to have a comparable number of transcripts to those found in our analysis [39]. Comparison of the anther transcriptomes revealed a high correlation between SAM, PMA, MA and SCP stages, indicating that there are subtle differences in expression across these three stages. The high similarity observed between SAM and PMA suggests that changes in the expression of very few genes are required to trigger anther development in rice. Most of the differentially expressed genes identified were found to be regulatory in nature, therefore, although these changes are few in number they can potentially initiate a chain of events in subsequent stages to influence expression of many downstream genes. It would seem that PMA is therefore a transitory phase, where the decision to undergo meiosis is taken in some specialized cells. PMA and MA represent very early stages of anther development in which there is relatively high representation of sporophytic tissue in comparison to the gametophytic tissue, with most of the transcriptome changes corresponding to sporogenous tissue and the developing tapetum. TPA, however, contains a relatively higher cellular mass that represents mature gametophytic tissue. In the current study, we clearly show marked differences in its transcript constitution from the rest of the anther transcriptomes investigated. The TPA stage is also characterized by the smallest and the most diverse transcriptome of the four stages analyzed. This could be by virtue of the distinctive transcriptomes of the male gametophyte and sperm cells [34] and down-regulation of a large number of genes that might not be required for the development of the gametophyte [39].

Comparison of our data with the recently published transcriptome of haploid male gametophyte development [40] substantiates our staging of anther development. Of the 188 unique probe-sets enriched in tricellular pollen (TCP) as identified by Wei and coworkers [40] 160 (85%) were expressed in TPA. Also of the 525 uni-nucleate microspore (UNM) enriched probe-sets, 405 (77%) were expressed in SCP.

In the present study, we have grouped differentially expressed genes into 10 co-expression groups. The information gathered from frequently co-expressed genes across multiple datasets and across different organisms has previously been used to verify gene interaction patterns and also to predict novel gene interaction networks [41-44]. A large number of genes (~34%) found in these co-expression groups have not even been annotated and even fewer of those that are annotated have been validated for their role in anther development. Therefore, identification of genes in these co-expression clusters pave the way for more focused investigations leading to a better understanding of gene regulatory networks.

The list of specifically activated transcription factors in PMA included four transcription factors (DRD1, ZOS2-03-C_2_H_2 _zinc finger, a helix-loop-helix, and a MYB transcription factor). Also, eleven genes (*OsMADS1, 2, 3, 4, 5, 6, 7, 8, 17, 34 *and *58*) belonging to the MADS-box family were up regulated by 2 folds in PMA with respect to SAM, which were also shown to be part of the pollen mother cell preferential transcriptome ([45], Additional File 6). Some of these MADS box genes have also been implicated in anther development [46,47]. Four members of the YABBY gene family were found to be specifically down regulated in PMA in comparison to SAM. However, in the MA, the NAM and AP2 class of genes dominated the list of differentially expressed transcription factors. While eight NAM and three AP2 family genes were up regulated in MA, eight AP2 genes were down regulated (Additional File 6). In SCP, besides a shuffling in the pool of transcription factors, down-regulation of the translation machinery was observed with more than 200 translation-related genes significantly affected by more than two folds. Most of these genes continued with the downward trend in TPA as well. In concurrence with previous observations from several groups [13,48,49] most of the down-regulated genes code for ribosomal proteins and elongation factors. There were also genes coding for LSM (like-Sm) domain containing and RNA recognition motif proteins that are known for their involvement in pre-mRNA processing [50,51].

A major proportion of the differentially expressed signal transduction components included those involved in calcium-mediated signaling, e.g. calcium dependent protein kinases, caleosins and other proteins containing C2 domain and EF hands. Genes involved in secondary metabolism (for example, those coding cytochrome 450, chalcone flavonone isomerase, strictosidinesynthase) showed marked up-regulation in MA and SCP. During meiosis and the single-celled microspore stage, tapetal cells are most active and are known to be involved in the synthesis of flavonoids and other secondary metabolites that eventually find their way to developing microspores [52], therefore, up-regulation of secondary metabolism related genes could in fact be related to tapetum development. Though not significant in number, genes involved in chromatin remodeling were also differentially expressed during progression of anther development. In addition, genes expressed in response to various abiotic stresses e.g., those coding for late embryogenesis abundant (LEA) proteins, dehydrins, and other senescence-associated proteins showed stage-specific differential expression, emphasizing that there may be parallels between the molecular mechanisms involved in reproductive development and stress (details of these genes can be found in Additional File 6).

Another interesting observation was that the anther transcriptome showed a high level of similarity with the seed transcriptome profile, when compared with other stages/tissues. The similarities in the two organs could be due to biochemical processes that are common to them. Both anthers and seeds are metabolically active tissues that exhibit high rates of cell division and both act as sinks for sugar derivatives, which are converted to starch at a rapid rate in these tissues. Furthermore, we have recently analyzed the similarities between reproductive developmental stages and dehydration stress in rice [53], where the findings indicate a high degree of overlap between genes that show differential expression during mature stages of panicle development, natural desiccation of seeds and plants exposed to dehydration stress. The findings in the present analysis highlight that both anthers and seeds could be utilizing similar regulatory networks for accumulation of starch, as they enter a phase of biological desiccation.

Gene regulation by means of RNA interference has been shown to play a vital role in anther development [54]. Reports have also shown the presence of functional miRNA in late stages of anther development [55]. Our data has also revealed up-regulation of genes coding for argonautes and other proteins with PAZ and PIWI domains in pre-meiotic and meiotic anthers in a stage preferential manner, suggesting that a different subset of RNAi machinery might be activated in reproductive tissues especially during early anther development. A rice pre-meiosis-specific argonaute gene *OsMEL1 *(LOC_Os03g58600) that has recently been implicated in male meiosis, [24] was shortlisted in our analysis and identified as being PMA-specific. It might therefore be interesting to explore the function of other similar components, determining whether there is reproduction-specific RNAi machinery.

### Towards understanding expression dynamics of the meiome

Meiosis has long been the subject of research with pioneering investigation in yeast (*Saccharomyces cerevisiae*) [56]. In flowering plants, identification of many meiotic mutants in *Arabidopsis*, rice and maize has led to functional characterization of close to fifty plant meiotic genes, that has substantially added to our current understanding of genes involved in plant meiosis [57,58]. The meiosis related genes characterized in rice include *PAIR1 *(*HOMOLOGOUS PAIRING ABERRATION IN RICE MEIOSIS1*) [59], *PAIR2 *[homologous to *Saccharomyces cerevisiae HOP1 *(*HOMOLOGOUS PAIRING 1*) and *Arabidopsis ASY1 *(*ASYNAPTIC1*)] [60], *OsRAD21-4 *(*RADIATION SENSITIVE 21-4*) [27], *OsDMC1 *(*DISRUPTION OF MEIOTIC CONTROL 1*) [61], *PAIR3 *[62], *OsMEL1*(*MEIOSIS ARRESTED AT LEPTOTENE1*) [24], *OsSDS *(*SOLO DANCERS*) *and OsRCK *(*ROCK-N-ROLLERS*) [63]. All these genes except *PAIR1, PAIR3 *and *OsMEL1 *were identified due to their homology to meiosis-related genes in yeast or *Arabidopsis*. However unlike in yeast and mammalian systems, we are still far from constituting the plant meiome.

**Figure 9:**
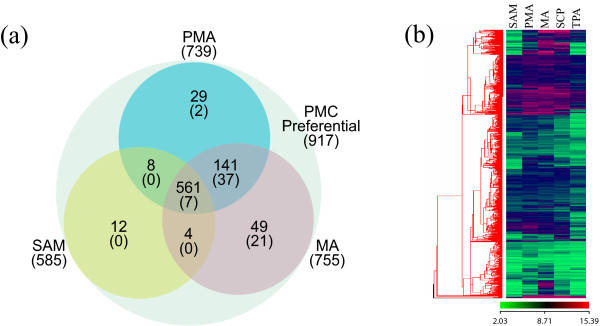
**Comparison of the pollen mother cell transcriptome with anther stages**. (a) Venn diagram showing the expression of pollen mother cell (PMC) preferential genes identified by Tang and co-workers [45] in PMA, MA and SAM. The number of probe-sets expressed in each stage is indicated, with the number of genes specifically expressed in anthers indicated in parentheses. (b) A heat-map representing the expression profiles of 702 PMC preferential genes (from the original 917 identified in comparing the 44K and 57K chip - *see reference *[45]*and the discussion*) that are expressed in SAM and the four stages of anther development (PMA, MA, SCP and TPA).

Our data shows that a large majority of rice meiosis homologues (from yeast and mammalian systems) do not express in a meiosis-specific manner. For example, *AtAHP2 *[64] is known to be involved in meiosis but it is expressed in other vegetative tissues as well. Likewise *AtRAD51 *is expressed in other stages but it has been shown to be essential for the progression of normal meiosis [65]. It could either mean that a greater proportion of genes involved in meiosis play a role in other cellular functions as well or that other genes may have taken up meiosis-specific functions in plants. Some meiotic genes have been shown to be plant-specific (for example, *Poor Homologous Synapsis*; *PHS1 *[66]). Recently, Tang and coworkers [45] carried out global expression profiling of laser-captured pollen mother cells (PMCs) in rice using the 44k Agilent array. By comparing the expression of PMC expressed genes to those expressed in seedlings and tricellular pollen they could identify 1,158 PMC-preferential genes. These PMC preferentially expressed genes contained many known meiotic genes, including *OsSPO11-1 *[67]*, PAIR1 *[59]*, PAIR2 *[60]*, PAIR3 *[62]*, OsDMC1 *[61]*, OsMEL1 *[24]*, OsRAD21-4 *[27]*, OsSDS *[63], and *ZEP1 *[31]. Since 917 of the 1158 PMC-preferential genes were represented in our data set (which is based on the Affymetrix 57k chip) we decided to analyze the expression profiles of these genes in all four stages of anthers (*see *Figure 9a; Additional File 8). Interestingly, of the 917 genes, 702 expressed both in pre-meiotic (PMA) and meiotic anthers (MA) (Figure 9b) and 561 of these expressed in SAM as well, albeit at relatively lower levels. However, when this data set of 917 genes was parsed through our data set of anther-specific genes, we could identify only 67 genes that were expressed in PMA and MA (44 were expressed in both PMA and MA, two were PMA-specific and 21 were expressed specifically in MA). Furthermore, most of the 702 genes that were expressed in both PMA and MA were also expressed at significant levels in other stages of anther development (Figure 9b). These observations strengthen our hypothesis that the expression for the majority of meiosis-related genes is not restricted to cells undergoing meiosis and that they may participate in functions other than meiosis or that other genes may have taken up meiosis-specific functions in plants. Therefore, the 372 genes constituting the meiotic anther specific expression profile (Figure 2) in this study should serve as a valuable resource for mining, the as yet unidentified, components of the meiotic machinery and associated regulatory networks in rice.

Differential expression analysis involving comparison of adjacent stages revealed an interesting pattern showing a steady and significant increase in the number of genes activating in post-meiotic stages (i.e., in SCP and TPA). In these stages a total of 4,232 transcripts were up regulated in comparison to 1,418 in PMA and MA combined. Additionally, the post-meiotic stages of SCP and TPA contained a significantly larger proportion of genes down regulated (6,395) when compared to the earlier stages of PMA and MA (968). A similar trend of a large number of genes getting transcriptionally activated and deactivated in post-meiotic anthers was also observed in maize [39]. In maize, 867 genes were up regulated while 908 were down regulated in post-meiotic stages of anther development. We detected orthologues for 345 and 346 genes, respectively, in our data set, and of these 265 (~77%) and 226 (~65%) exhibited similar expression profiles in rice. Incidentally, a large percentage of the down-regulated genes in both rice and maize anthers are those that are expressed at significant levels (normalized average expression value ≥ 50) (data not shown), which suggests that meiosis may act as a two-way molecular switch that activates a large number of gametophytic genes, and at the same time, shuts down the sporophytic machinery that is presumably not necessary for male gametophyte development.

### Sperm cell transcriptome

The transcriptomes of pollen and sperm cells have been reported to be smaller than and distinct from those of vegetative tissues [34,35]. By comparing known sperm cell transcriptomes with the TPA transcriptome, we have attempted to identify the constitution of the rice sperm cell transcript pool. We show that a large proportion of transcripts constituting the *Arabidopsis *sperm cell (82.3%), maize sperm cell (90.1%) and the lilly generative cell (86.7%) transcriptomes were represented in the rice TPA transcriptome, suggesting a high degree of similarity between sperm cell transcriptomes of monocot and dicot plants. Categorization of TPA activated and up-regulated genes into GO functional groups (Figure 6) indicates that genes encoding signal transduction components, cell structure components, transporters, transcription factors and stress related pathways, could be the major contributors to the sperm cell transcriptome. Since 448 of the TPA expressed genes have not been previously reported to be expressed in vegetative tissues and throughout the stages of seed development, they could serve as a useful resource to mine putative sperm cell transcripts for validation of their function in this unique cell type.

## Conclusions

### Implications in defining components of biochemical and gene regulatory networks

Identification of co-expressing clusters in a developmental event is indicative of common or related regulatory pathways. Co-expression is often related to co-regulation, and genes that follow similar expression profiles may be the targets of the same transcription factors. Our studies have allowed identification of specifically regulated genes. Comparison with four of the vegetative stages and five seed development stages of rice has allowed segregation of transcripts dedicated to anther development function, especially the three co-expression groups that identify genes showing expression peaks in the four stages of anthers (Figure 2). Many of the genes in these clusters have not yet been annotated and as such, deserve further attention as a source of genes that could have (as yet) unidentified roles in meiosis and other stages examined. Such analysis is of great significance for future research, with several candidates now being targeted for studies that will build towards our understanding of regulatory networks and validating gene function(s).

## Authors' contributions

PD carried out the microarray experiments for PMA, SCP and TPA stages, performed *in situ *hybridization experiments, analyzed the data and drafted the manuscript. RS identified the anther stages and performed the microarray experiment for MA stage. WDB and JAA helped in the data analysis and preparation of the manuscript. SK conceptualized and designed the experiments, contributed to the data analysis and manuscript writing. All authors read and approved the final manuscript.

## Supplementary Material

Additional File 1 **Box-Whisker plot showing the range of expression of *Magnoporthe *genes across the 14 stages of development used in the microarray**. LM, Mature Leaf; LY, Y-Leaf; R, Root; SDL, 7 day old seedling; SAM, Shoot apical meristem; PMA, Pre-meiotic anther; MA, Meiotic anther; SCP, Anthers with single celled pollen; TPA, Anther with trinucleate pollen; S1-S5, Seed stages from 0 days after pollination (DAP) till 30 DAP. Click here for file

Additional File 2 **List of rice homologues for *Arabidopsis*, lily and maize sperm cell transcripts and significance values from BLASTx searches and their comparison with the TPA transcriptome**. Click here for file

Additional File 3 **List of Primers used in Real-time PCR**. Click here for file

Additional File 4 **Scatter plots comparing gene expression of four stages of anther development as well as shoot apical meristem (SAM)**. Numerical figures in the blocks show the number of genes with at least 2-fold differential expression between the stages. The correlation co-efficient for gene expression between the stages is indicated at the top of each plot. Clearly, PMA (pre-meiotic anther), MA (meiotic anther) and SCP (single-celled pollen) have more similarity in their transcriptome than TPA (tri-nucleate pollen), which shows higher variation in transcripts. Click here for file

Additional File 5 **MAS5 detection calls and p-values for the list of unique probe-set IDs and probe-set lists of SAM, PMA, MA, SCP, seed, and leaf transcriptomes**. Click here for file

Additional File 6 **Raw and log**_**2 **_**transformed expression values, probe set IDs, Locus IDs, functional categories, putative functions and cluster categorization of 11,915 genes differentially expressed in anthers**. Click here for file

Additional File 7 **Expression profiles of early meiosis genes in yeast**. Numbers on the Y axis are normalized. The data was normalized for the minimum value as zero. Meiotic time points are shown on the X axis. Gene names written in red are at least 1.95-fold up regulated when comparing the maximum value of meiotic verses maximum value of the non-meiotic stages. Fold changes are shown in parentheses. The expression data was obtained from GEO accession number GSE18181. Click here for file

Additional File 8 **A comparison of the pollen mother cell transcriptome with that of SAM, PMA, MA, SCP and TPA**. Click here for file
