# Supplementary figures and images for: Analysis of anther transcriptomes to identify genes contributing to meiosis and male gametophyte development in rice
Source: BMC Plant Biol. 2011 May 9;11:78. doi: 10.1186/1471-2229-11-78 (PMC3112077; doi:10.1186/1471-2229-11-78)

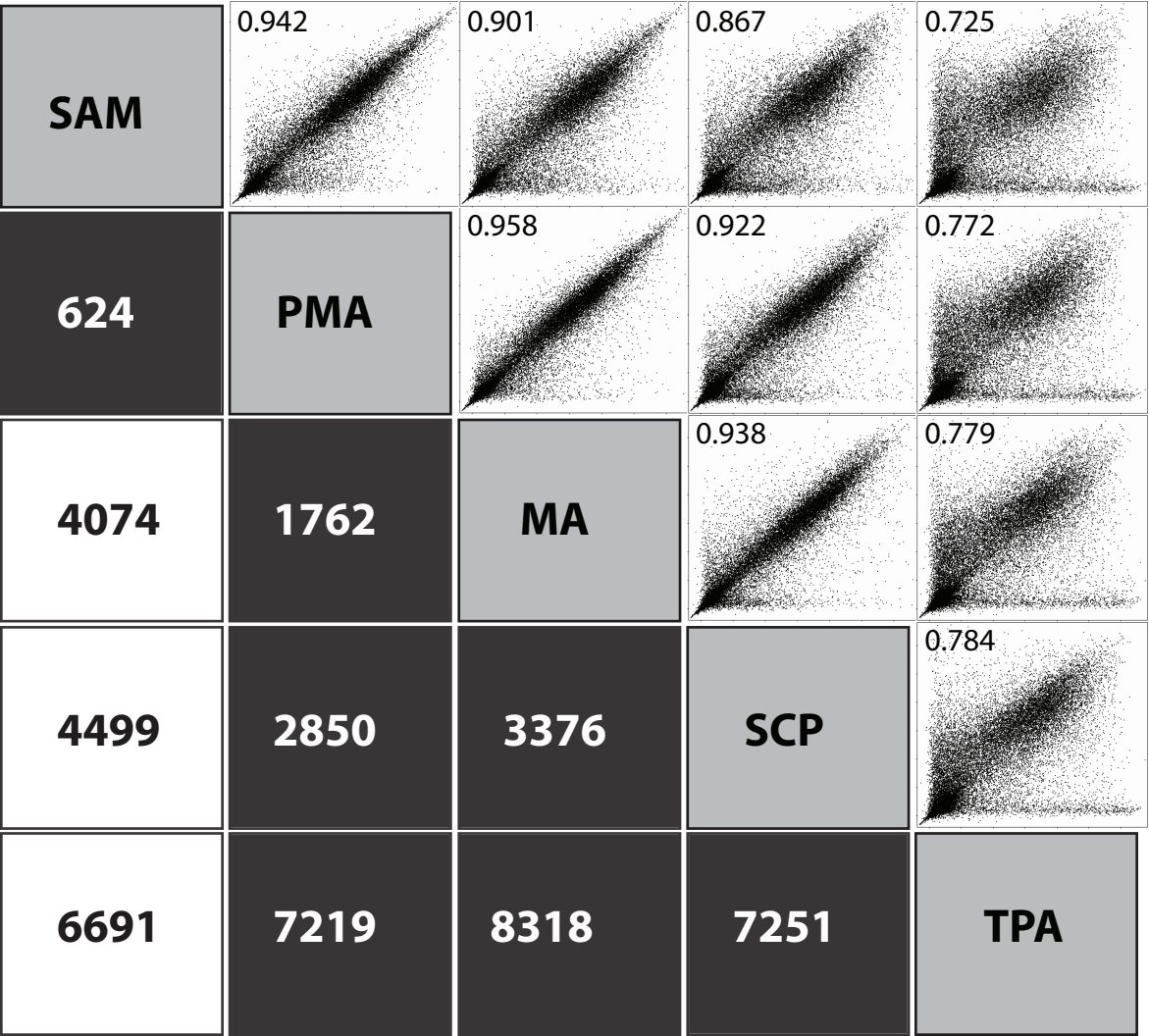

Supplement: Additional File 4 — Scatter plots comparing gene expression of four stages of anther development as well as shoot apical meristem (SAM). Numerical figures in the blocks show the number of genes with at least 2-fold differential expression between the stages. The correlation co-efficient for gene expression between the stages is indicated at the top of each plot. Clearly, PMA (pre-meiotic anther), MA (meiotic anther) and SCP (single-celled pollen) have more similarity in their transcriptome than TPA (tri-nucleate pollen), which shows higher variation in transcripts. [file 1471-2229-11-78-S4.PDF]
